# *VEGFA* rs2010963 GG genotype is associated with superior adaptations to resistance *versus* endurance training in the same group of healthy, young men

**DOI:** 10.1007/s00438-022-01965-4

**Published:** 2022-11-03

**Authors:** Maxime Boidin, Ellen A. Dawson, Dick H. J. Thijssen, Robert M. Erskine

**Affiliations:** 1 School of Sport and Exercise Sciences, Liverpool John Moores University, Byrom Street, Liverpool, L3 3AF UK; 2 Liverpool Centre for Cardiovascular Science, Liverpool, UK; 3 Department of Sport and Exercise Sciences, Institute of Sport, Manchester Metropolitan University, Manchester, UK; 4 Research Institute for Health Sciences, Department of Physiology, Radboud University Medical Center, Nijmegen, The Netherlands; 5 Institute of Sport, Exercise and Health, University College London, London, UK

**Keywords:** Aerobic training, Strength training, Genetic variation, Training response, Maximal force

## Abstract

**Purpose:**

We used a within-subject, cross-over study to determine the relationship between the intra-individual adaptations to four weeks’ resistance (RT) *versus* four weeks’ endurance (END) training, and we investigated whether three single nucleotide polymorphisms (SNPs) were associated with these adaptations.

**Methods:**

Thirty untrained, healthy, young men completed a cycling test to exhaustion to determine peak oxygen uptake (V̇O_2peak_), and a knee extension (KE) maximum voluntary isometric contraction (MVIC) of the right leg before and after four weeks’ supervised RT (four sets of 10 repetitions at 80% single repetition maximum unilateral KE exercise, three times weekly) and four weeks’ supervised END (30 min combined continuous/interval cycling, three times weekly), separated by a three-week washout phase. Participants were genotyped for the *ACTN3* rs1815739, *NOS3* rs2070744 and *VEGFA* rs2010963 SNPs.

**Results:**

The intra-individual adaptations regarding percentage changes in MVIC force and V̇O_2peak_ following RT and END, respectively, were unrelated (*r*^2^ = 0.003; *P* = 0.79). However, a *VEGFA* genotype × training modality interaction (*P* = 0.007) demonstrated that *VEGFA* GG homozygotes increased their MVIC force after RT (+ 20.9 ± 13.2%) more than they increased their V̇O_2peak_ after END (+ 8.4 ± 9.1%, *P* = 0.005), and more than *VEGFA* C-allele carriers increased their MVIC force after RT (+ 12.2 ± 8.1%, *P* = 0.04). There were no genotype × training modality interactions for the *ACTN3* or *NOS3* SNPs.

**Conclusion:**

High/low responders to RT were not consequently high/low responders to END or vice versa. However, preferential adaptation of *VEGFA* rs2010963 GG homozygotes to RT over END, and their greater adaptation to RT compared to *VEGFA* C-allele carriers, indicate a novel genetic predisposition for superior RT adaptation.

## Introduction

Short-term (*e.g.* three to five weeks) resistance training (RT) is a potent stimulus for increasing muscle strength (Moritani and deVries 1979; Seynnes et al. 2007; Buckthorpe et al. 2015), while short-term endurance training (END) increases peak oxygen uptake (V̇O_2peak_) (Dunham and Harms 2012; Murias et al. 2016; Dawson et al. 2021). However, until relatively recently, studies focussed on the mean adaptation to these chronic exercise modalities, while variation around the mean was primarily attributed to measurement error. It has since become apparent that the variable response to RT (Hubal et al. 2005; Erskine et al. 2010a) and END (Bouchard et al. 1999) cannot be simply explained by measurement error alone, and understanding this variation may in fact elucidate novel mechanisms controlling gains in muscular strength, as well as cardiovascular fitness. However, it is not yet known if the variable adaptations to dichotomous modalities of short-term exercise training, *i.e.* RT and END, are related within individuals. In other words, are high responders to short-term RT (in terms of strength gains) likely to show a poor response to END (in terms of V̇O_2peak_) or vice versa. Alternatively, will high responders to one mode of chronic exercise also respond positively to the other mode of exercise? A recent cross-over training study found no relationship between strength gains following 12 weeks’ RT and changes in V̇O_2peak_ following 12 weeks’ END (Marsh et al. 2020). However, this study incorporated multiple types of RT (*e.g.* upper- and lower-limb) and END (*e.g.* running and cycling) of varying intensities and volume. Such an approach may introduce overlap in the effects of both exercise modalities, and therefore make it difficult to directly compare the effects of RT with END in the same group of individuals. More specific training modalities and assessments would improve our understanding of the physiological mechanisms underpinning the adaptations to dichotomous modes of short-term chronic exercise, while providing evidence to support or refute the case for personalised exercise prescription to optimise the health benefits of exercise.

Furthermore, the variable response to both RT (Thomis et al. 1998) and END (Bouchard et al. 1999) in previously untrained individuals is known to have significant heritability, *i.e.* the genetic component is suggested to explain ~ 20% of the change in strength and ~ 47% of the change in V̇O_2peak_. Moreover, there is evidence that variation within specific genes (*e.g.* single nucleotide polymorphisms, SNPs) influence the adaptation to chronic exercise, although this evidence is based on single modality exercise programmes. For example, the α-actinin-3 (*ACTN3*) gene encodes the α-actinin-3 protein that anchors actin to the Z-disk in human type II skeletal muscle fibres and plays an instrumental role in hindering the conversion of the larger, stronger, faster type II fibre characteristics to smaller, weaker, more oxidative type I fibre properties (Seto et al. 2013). Some studies (Delmonico et al. 2007), but not all (Clarkson et al. 2005; Erskine et al. 2014), suggest that *ACTN3* rs1815739 RR homozygotes demonstrate greater improvements in maximal strength and power following chronic RT, while there is evidence that *ACTN3* rs1815739 XX homozygotes demonstrate greater improvements in V̇O_2peak_ following END [see review of Del Coso et al. (2019)]. However, no study has yet investigated if this SNP may help to explain potential intra-individual variability in the adaptations to RT *and* END.

Another gene of interest regarding the variable response to chronic exercise is the nitric oxide (NO) synthase 3 (*NOS3*) gene, which encodes endothelial NO synthase (eNOS) (Marsden et al. 1993), and is related to vascular health (Zmijewski et al. 2018). For example, systolic and diastolic blood pressure have been shown to decrease after 12 weeks’ END only in *NOS3* rs2070744 TT homozygotes and not C-allele carriers (Trapé et al. 2021), suggesting that *NOS3* TT genotype influences a positive vascular adaptation to END. Furthermore, we have recently shown that *NOS3* rs2070744 TT homozygotes improved their vascular function after END, but not after RT (Dawson et al. 2021). *NOS3* C-allele carriers, on the other hand, showed a tendency to improve vascular function following RT but not END (Dawson et al. 2021), thus providing evidence that a genetic association with the adaptation to chronic exercise training is specific to the mode of exercise. The T-allele has also been linked to elite power athlete status and superior sprint/power performance (Murtagh et al. 2020), suggesting it may be associated with superior adaptations to RT over END within the same group of individuals, although this has yet to be investigated.

A third gene that may influence the intra-individual adaptations to RT and END in humans is the vascular endothelial growth factor-A (*VEGFA*) gene, which encodes the VEGF-A protein that regulates erythropoiesis, angiogenesis, and muscle blood flow (Dai and Rabie 2007). Specific SNPs (e.g. rs2010963) within the *VEGFA* gene have been associated with endurance performance in humans (Prior et al. 2006) and with an increase in capillary density following END in mice (Booth et al. 2015) but not RT in humans (Campos et al. 2002). However, no study has investigated whether variations of this gene are associated with the intra-individual adaptations to RT and END in humans. In fact, it is not yet known if variation in any genes can help explain differences in the adaptations (with respect to strength gains and improvements in cardiovascular fitness) to RT and END within the same group of individuals.

We therefore used a within-subject, cross-over design to determine the relationship between the adaptations to four weeks’ RT (in terms of strength gains) *versus* four weeks’ END (in terms of changes in V̇O_2peak_) within the same group of previously untrained, healthy, young men. Further, we investigated whether SNPs within the *ACTN3*, *NOS3*, and *VEGFA* genes were associated with intra-individual differences in the adaptations to RT *versus* END. We hypothesised that individuals carrying at least one *ACTN3* rs1815739 R-allele (RR + RX genotypes), and those homozygous for the *NOS3* rs2070744 T-allele (TT genotypes) would demonstrate greater RT than END adaptations, and that these adaptations would be the opposite to those demonstrated by their *ACTN3* XX homozygote and *NOS3* C-allele carrying counterparts. We also hypothesised that carriers of the *VEGFA* rs2010963 C-allele would adapt better to END than RT, while GG homozygotes would demonstrate superior adaptations to RT than END.

## Methods

### Study design and participant recruitment

Forty previously untrained (but recreationally active), healthy, young men from the student population at Liverpool John Moores University volunteered to take part in this study. A minimal sample size was estimated a priori with G*Power software (v3.1.9.6, Heinrich-Heine-Universität Düsseldorf, Düsseldorf, Germany). The estimation was performed using a small-to-medium effect size (*ƞ*_p_^2^ = 0.05; *α* = 0.05; power = 0.80), based on the RT-induced peak power gains in male *ACTN3* rs1815739 RR *vs.* XX homozygotes in the study by (Delmonico et al. 2007). A minimum of 28 participants was deemed necessary to detect a genotype × exercise modality interaction effect. However, due to the relatively demanding nature of the study design (*e.g.* frequency of training sessions and overall study duration), we expected ~ 20% participants would drop out, so we increased our initial sample size to *n* = 40. Indeed, as anticipated, 10 participants withdrew due to personal reasons at various stages of the study, and 30 participants (height: 1.79 ± 0.07 m; body mass: 77.0 ± 9.7 kg; age: 21 ± 2 years) completed the study. Volunteers with history of lower-limb musculoskeletal injuries or cardiovascular disease, or who reported cardiovascular risk factors or were using any medication or nutritional supplementation that could potentially affect physical performance, or who smoked, were excluded from the study. All participants gave written informed consent before taking part in the study, which was approved by Liverpool John Moores University Research Ethics Committee (approval number: 13/APS/032) and adhered to the Declaration of Helsinki.

### Experimental design

**Fig. 1:**
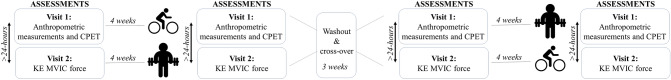
Experimental design of the study. *CPET* cardiopulmonary exercise testing, *MVIC* maximum voluntary isometric contraction

**Fig. 2:**
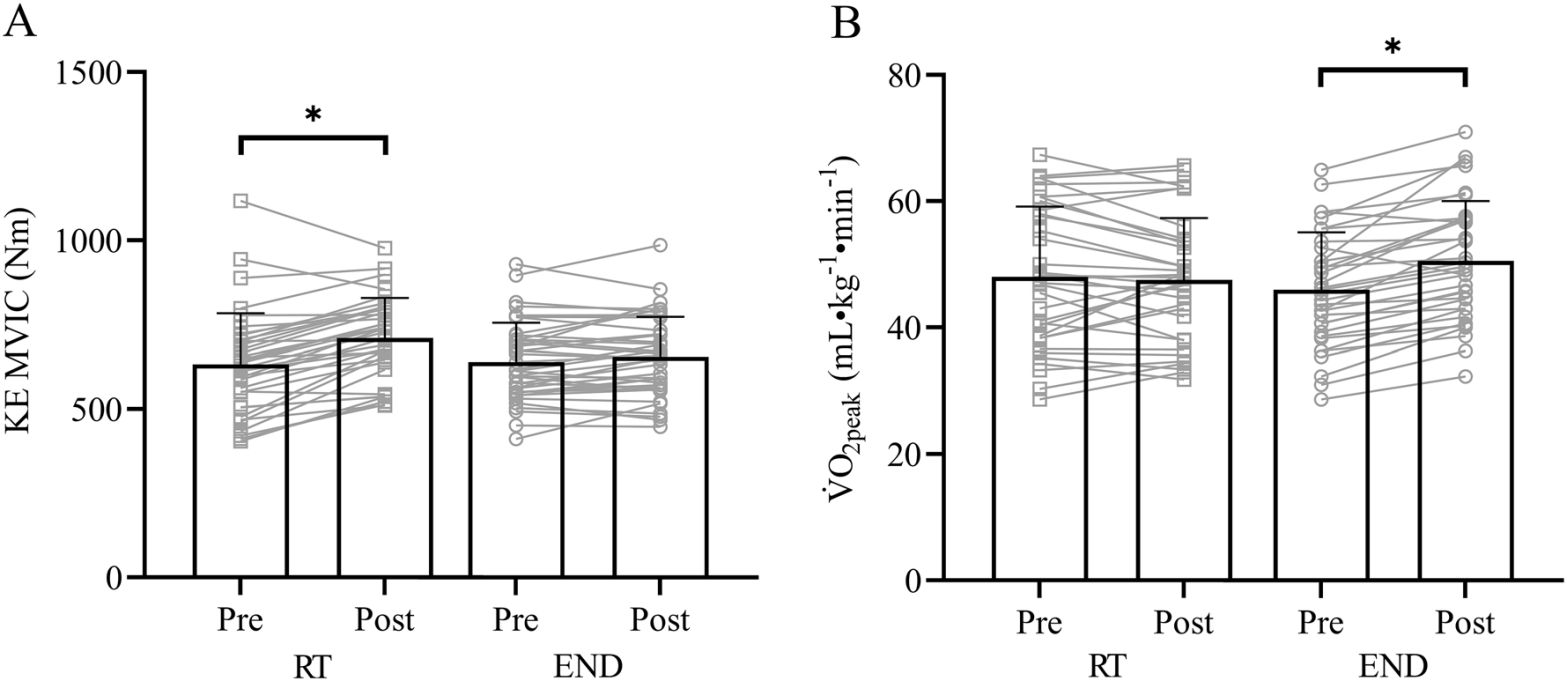
Resistance (RT) and endurance (END) exercise training-induced changes (pre to post-RT and END) in knee extensor (KE) maximum voluntary isometric contraction (MVIC) force (A) and peak oxygen uptake (V̇O_2peak_) (B); *P < 0.05 post hoc paired *t*-test analyses (pre to post RT or END)

Before and after both four-week exercise training programmes, all participants reported to the laboratory on two occasions to undergo testing procedures, separated by at least 24 h between visits. During the first visit, all underwent anthropometric measurements and a cycling test to exhaustion with gas exchange analysis to determine V̇O_2peak_, and all provided a venous blood sample, from which DNA was isolated for genotyping. During the second visit, knee extenstion (KE) maximum voluntary isometric contraction (MVIC) force of the right leg was measured. This order was kept the same throughout the entire protocol. Participants completed 12 sessions over a four-week period, either RT or END training in a randomised, balanced cross-over design (Fig. 1). Detraining adaptations (following either END or RT) seem to follow a similar time course to that of training adaptations in young, untrained individuals, *i.e.* three to four weeks’ END or RT elicit a 10–15% *increase* in either V̇O_2peak_ or MVIC force, while similar *reductions* in these variables are observed after three to four weeks’ detraining (Narici et al. 1989; Neufer 1989). Thus, in between each four-week END or RT training block, our participants completed a washout period of three weeks to ensure V̇O_2peak_ or MVIC force had returned to baseline prior to the next four-week training period (Fig. 2). For each participant, all testing at baseline, after the first four weeks’ training block, after the three-week washout period, and after the second four weeks’ training block were completed within a seven-day period of the first/last training session.

### Measurements

*Cardiopulmonary exercise testing* (CPET) was performed in all participants on an electronically braked cycle ergometer (Daum-electronic premium, 8i ergo-bike, Fürth, Germany). V̇O_2peak_ (mL∙kg^−1^∙min^−1^), and respiratory exchange ratio (RER) were measured continuously at rest and during exercise using a metabolic system (Metamax 3B, MM3B, Cortex, Leipzig, Germany). Measurements also included power output (Watts, W), heart rate (HR, beats per minute, bpm) with a Polar FT1 heart rate monitor with a Pro chest strap Strap (Polar Electro Oy, Kempele, Finland). The incremental protocol began with a power output of 95 W, followed by an increase of 35 W every 3 min until exhaustion, while maintaining a cadence of 80 rpm. The oxygen uptake (V̇O_2_) was considered to have peaked (V̇O_2peak_) when at least two of the three following criteria were met: (i) a levelling off of V̇O_2_ with increasing intensity (an increase of no more than 2 ml∙kg^−1^∙min^−1^); (ii) a HR within 10 beats∙min^−1^ of the age-predicted maximum (220 minus age in years); (iii) a respiratory exchange ratio (RER) greater than 1.05. V̇O_2peak_ was determined from the mean of the three consecutive highest values over a 30-s interval reached during the last stage of the protocol. Strong verbal encouragement was given throughout the test. This was followed by 15 min’ unloaded recovery cycling at a self-selected cadence.

#### Knee extension MVIC force

Participants were secured with inextensible straps to in an isokinetic dynamometer (IKD) chair (Lido Active, Loredan, Davis, CA, USA), with a hip angle of 90° (180° = supine) and a 90° knee flexion angle (0° = full knee extension). The right ankle was connected to the force transducer (KAP, Bienfait B.V. Haarlem, The Netherlands) and the force signal was interfaced with an analog-to-digital converter (MP150, Biopac Systems, CA, USA), sampled at 2000 Hz with a PC using Acqknowledge software (version 5, Biopac) and low-pass filtered (10-Hz). After a warm-up of 10 submaximal voluntary contractions, with participants increasing their force output each time, participants were instructed to perform two MVICs, separated by a one-minute rest period. If the second MVIC was > 5% higher than the first, a third attempt was performed until the highest MVIC was < 5% higher than the next highest. Verbal encouragement was provided by researchers and biofeedback was provided to participants by projecting their MVIC attempts on a screen in front of them in real time.

### DNA extraction and genotyping

A blood sample was drawn into a 10-mL EDTA vacutainer (BD Vacutainer Systems, Plymouth, UK) from a superficial forearm vein. The whole blood was aliquoted into 2-mL tubes (Eppendorf AG, Hamburg, Germany) and stored at −80 °C until subsequent analysis. DNA purification from whole blood samples was performed manually using a QIAamp DNA Blood Mini Kit (Qiagen Ltd., Manchester, UK), following the manufacturer’s guidelines, as described previously (Erskine et al. 2012). Samples of DNA were then stored at 4 °C until subsequent genotyping. Real-time polymerase chain reaction was performed (Rotor-Gene Q, Qiagen) to establish the genotypes of the *ACTN3* rs1815739, *NOS3* rs2070744, and *VEGFA* rs2010963 SNPs for each participant. Each 10 *μ*L reaction volume contained 5 *μ*L Genotyping Master Mix (Applied Biosystems, Foster City, USA), 3.5 *μ*L nuclease-free H_2_O (Qiagen), 0.5 *μ*L SNP (*ACTN3*, *NOS3* or *VEGFA*) TaqMan genotyping assay (Applied Biosystems), plus 1 *μ*L DNA sample. Both negative [1 *μ*L nuclease-free H_2_O (Qiagen) replaced the DNA template] and positive controls were included in each RT-PCR run, which used the following protocol: denaturation at 95 °C for 10 min, followed by 40 to 50 cycles of incubation at 92 °C for 15 s, then annealing and extension at 60 °C for 1 min. Genotypes were determined using Rotor-Gene Q Pure Detection 2.1.0 software (Qiagen). All samples were analysed in duplicate and there was 100% agreement between genotypes calls for samples from the same participant.

### Exercise training

#### Resistance training

All participants completed all 12 training sessions, which were supervised by members of the research team and were performed three times a week for four weeks. The RT was performed on a leg extension machine (Technogym, Gambettola, Italy) by alternating one leg at a time. Prior to the first training session of each week, the leg extension single repetition maximum (1-RM) was assessed to determine the training load for that week and ensure the load was progressively increased (each week, the training load was adjusted according to the new 1-RM) (Haff et al. 2015). Before each RT session, a warm-up set of 10 repetitions at 40% 1-RM was performed, which was followed by 4 sets of 10 repetitions at 80% 1-RM for each leg, with 2 min recovery between sets.

#### Endurance training

All participants completed all 12 training sessions, which were supervised by members of the research team and were performed three times a week for four weeks. Each 30 min END session comprised cycling on a cycle ergometer (Lode BV, Groningen, the Netherlands), with intensity progressively increased on a weekly basis. Week 1 comprised steady-state (SS) cycling at 70% maximal HR (HR_max_, assessed during the CPET); week 2 comprised SS at 70% HR_max_ interspersed every sixth min with 1 min at 90% HR_max_; week 3 comprised SS at 80% HR_max_; week 4 comprised SS at 80% HR_max_ interspersed every sixth min with 1 min at 90% HR_max_. Before and after each training session, participants performed a 3-min warm-up/cool-down at 60–80 W.

### Statistical analysis

Data are presented as mean ± standard deviation. The statistical analyses were performed with GraphPad Prism 9.0.1 (GraphPad Software, Inc., La Jolla, California, USA). Differences were defined as statistically significant when *P* < 0.05. After ensuring a normal distribution, bivariate Pearson’s correlations were used to determine relationships between intra-individual percentage changes in MVIC force after RT and percentage changes in V̇O_2peak_ after END. A one-way analysis of variance (ANOVA) was used to compare baseline values between the two exercise modalities. A two-way ANOVA with repeated measures (exercise modality × time) was used to compare body mass, blood pressure, and heart rate between the two training interventions. Genotype frequency distributions for all three SNPs were tested for compliance with Hardy–Weinberg Equilibrium using Pearson’s *χ*^2^ tests. A two-way mixed ANOVA was used to compare percentage change in MVIC force after RT with percentage change in V̇O_2peak_ after END between genotypes for each SNP: *ACTN3* (RR + RX *vs.* XX), *NOS3* (CC + TC *vs.* TT), and *VEGFA* (CC + GC *vs.* GG). In the case of a significant two-way interaction, post hoc paired *t *tests were used to determine the preferential exercise adaptation within-genotype, while post hoc independent *t *tests were used to determine the preferential exercise adaptation between genotypes. Effect sizes for the two-way mixed ANOVA interactions (partial eta-squared, *η*_p_^2^), and *t *tests (Cohen’s *d*) were reported for each statistical model. The thresholds of Cohen’s *d* and *η*_p_^2^ are defined as small (*d* = 0.20 and *η*_p_^2^ = 0.01), medium (*d* = 0.50 and *η*_p_^2^ = 0.06) and large (*d* = 0.80 and *η*_p_^2^ = 0.14) (Cohen 1988; Bakeman 2005). A false discovery rate (FDR) of 10% was used to control for multiple comparisons (Benjamini and Hochberg 1995).

## Results

When categorised according the genotype groups for each SNP, none of the baseline characteristics (Table 1) differed between groups, except for age between *NOS3* CC + TC and TT genotypes (*P* = 0.02). Body mass, blood pressure and resting heart rate did not change after either RT or END. No order effect for training modality was found for either MVIC force or V̇O_2peak_ (*P* > 0.05). The genotype and allele frequency distributions for the three SNPs were as follows:

**Table 1:** Baseline characteristics of the young healthy untrained males according the *ACTN3* rs1815739, *NOS3* rs2070744, and *VEGFA* rs2010963 genotypes

	Genotypes		*P*-value
	*ACTN3*		
	RR + RX (*n* = 22)	XX (*n* = 8)	
Age, years	20 ± 2	22 ± 4	0.09
Height, cm	178.9 ± 6.8	178.4 ± 6.6	0.86
Body mass, kg	75.7 ± 8.4	81.6 ± 14.9	0.18
BMI, kg∙m^−2^	23.8 ± 2.5	25.6 ± 3.4	0.13
	*NOS3*		
	CC + TC (*n* = 18)	TT (*n* = 12)	
Age, years	22 ± 3	19 ± 2	0.02*
Height, cm	179.0 ± 5.6	178.4 ± 8.3	0.78
Body mass, kg	78.3 ± 11.4	75.1 ± 8.3	0.41
BMI, kg∙m^−2^	24.5 ± 3.1	23.6 ± 2.2	0.37
	*VEGFA*		
	CC + GC (*n* = 15)	GG (*n* = 15)	
Age, years	20 ± 2	21 ± 3	0.62
Height, cm	176.2 ± 6.7	180.1 ± 6.3	0.12
Body mass, kg	74.8 ± 8.4	78.3 ± 11.1	0.37
BMI, kg∙m^−2^	24.1 ± 2.4	24.2 ± 3.0	0.89

Variables are expressed as mean ± SD

*BMI* Body mass index

*P *values represent the unpaired *t* test analysis. *P < 0.05

*ACTN3* rs1815739 genotype: RR, *n* = 9 (0.30); RX, *n* = 13 (0.43); XX, *n* = 8 (0.27); allele: *R*, 0.52; *X*, 0.48;

*NOS3* rs2070744 genotype: TT, *n* = 12 (0.40); CT, *n* = 14 (0.47); CC, *n* = 4 (0.13); allele: *T*, 0.63; *C*, 0.37;

*VEGFA* rs2010963 genotype: GG *n* = 15 (0.50), GC *n* = 13 (0.43), CC *n* = 2 (0.07); allele: *G*, 0.72; *C*, 0.28.

Genotype frequency distributions for each SNP were in Hardy–Weinberg Equilibrium (*χ*^2^ ≤ 0.535; *P* ≥ 0.765).

### Between-exercise modality comparison

We found a time × exercise modality interaction for knee extensor MVIC and V̇O_2peak_ (*P* < 0.001 for both) with a greater improvement of knee extensor MVIC force after RT and a greater improvement of V̇O_2peak_ after END after Bonferroni post hoc analyses (*P* < 0.001). Post hoc paired *t *tests demonstrated that RT improved MVIC force (*P* < 0.001), but not V̇O_2peak_ after the four-week training intervention (Fig. 2A). Similarly, END improved V̇O_2peak_ (P < 0.001), but not MVIC force (Fig. 2B). Paired *t *tests confirmed that V̇O_2peak_ (pre-END *vs.* pre-RT, *P* = 0.20) and MVIC force (pre-RT *vs.* pre-END, *P* = 0.76) had returned to baseline values after the three-week washout period.

### Within-individual comparison

**Fig. 3:**
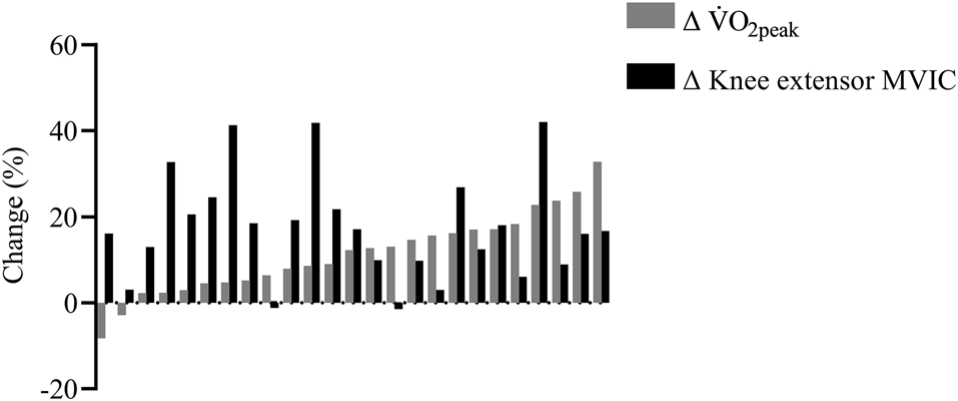
Individual percentage changes in knee extensor maximum voluntary isometric contraction (MVIC) force after resistance training (RT) compared to change in peak oxygen uptake (V̇O_2peak_) after endurance training (END) in all 30 individuals

When comparing the individual percentage change in knee extensor MVIC force (Fig. 3) after RT *versus* respective changes in V̇O_2peak_ after END in all 30 participants, we observed large intra- and inter-individual variation. However, the individual changes in MVIC force after RT did not correlate with the changes in V̇O_2peak_ after END (r^2^ = 0.003; P = 0.79).

### ACTN3 rs1815739 genotype

**Fig. 4:**
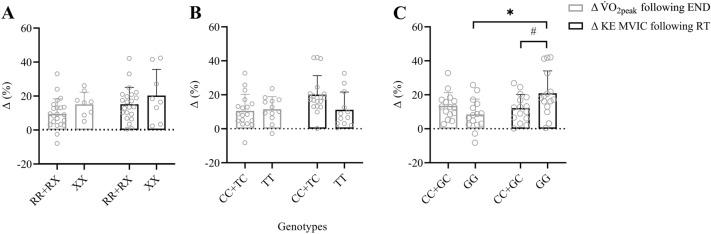
Percentage changes in knee extensor maximum voluntary isometric contraction (MVIC) force after resistance training (RT) compared to change in peak oxygen uptake (V̇O_2peak_) after endurance training (END) in individuals of *ACTN3* rs1815739 RR + RX and XX (**A**), *NOS3* rs2070744 CC + TC and TT (**B**), and *VEGFA* rs2010963 CC + GC and GG (**C**) genotypes; *within-genotype difference (*P* < 0.05); # between-genotypes difference (*P* < 0.05)

No significant genotype × exercise modality interaction was found for the change in MVIC force and V̇O_2peak_ following RT and END, respectively (*P* = 0.93, *η*_p_^2^ < 0.001, Fig. 4A). We found a tendency for a main effect of *ACTN3* genotype regarding a training-induced change in MVIC force and V̇O_2peak_, with XX homozygotes tending to display larger changes in MVIC force and V̇O_2peak_ compared to R-allele carriers (RR + RX genotypes) (*P* = 0.07, Fig. 4A).

### NOS3 rs2070744 genotype

No main effects for genotype or training modality were found (Fig. 4B). We found a tendency for a *NOS3* genotype × exercise modality interaction (*P* = 0.07, *η*_p_^2^ = 0.05), where percentage change in MVIC force was greater in CC + TC genotype following RT compared to the change in V̇O_2peak_ in CC + TC (post hoc, P = 0.01, d = 0.84) and TT (post hoc, *P* = 0.03, *d* = 0.77) genotypes following END, and compared to the change in MVIC force in TT homozygotes following RT (post hoc, *P* = 0.04, Fig. 4B).

### VEGFA rs2010963 genotype

There was a *VEGFA* genotype × exercise modality interaction (*P* = 0.007, *η*_*p*_^2^ = 0.09) regarding percentage changes in MVIC force following RT and V̇O_2peak_ after END, and a main effect for exercise modality (*P* = 0.03) (Fig. 4C). Post hoc *t *tests showed that the percentage change in MVIC force following RT was greater than the change in V̇O_2peak_ following END in GG homozygotes only (*i.e.* intra-genotype differences in the adaptation to RT *vs*. END, paired *t*-test, *P* = 0.005, *d* = 0.98). Post hoc *t *tests showed that MVIC force improved after RT in both CC + CG (paired *t*-test, *P* < 0.001, *d* = 0.60) and GG (paired *t*-test, *P* < 0.001, *d* = 0.70) genotypes but the change in MVIC force was greater in GG homozygotes compared to CC + CG genotypes (independent *t*-test, *P* = 0.04, *d* = 0.75), and tended to be greater than the change in V̇O_2peak_ after END in CC + CG genotypes (independent *t*-test, *P* = 0.07, *d* = 0.66). Further post hoc *t *tests revealed that V̇O_2peak_ improved after END in both CC + CG (*P* < 0.001, *d* = 0.55) and GG (*P* = 0.002, *d* = 0.41) genotypes, with no difference between both genotypes (*P* = 0.11, *d* = 0.59). Furthermore, a post hoc power analysis revealed that this *VEGFA* genotype × exercise modality interaction had a power of 0.823.

## Discussion

To the best of our knowledge, this work represents the first cross-over design study in humans, which directly compares the effects of short-term (*i.e.* four weeks’) endurance (END) *versus* resistance (RT) exercise training on cardiovascular fitness and muscle strength in the same group of individuals, and which subsequently links the intra-individual variability in the adaptations to RT *vs*. END to genetic variation. Although intra-individual adaptations to RT and END were unrelated (Fig. 3), *VEGFA* rs2010963 GG homozygotes adapted better to RT (in terms of strength gains) than they did to END (in terms of V̇O_2peak_ improvements). *VEGFA* GG homozygotes also increased their MVIC force more than *VEGFA* C-allele carriers after RT. Thus, we have provided evidence that *VEGFA* rs2010963 genotype is associated with both intra- and inter-individual variation in the adaptation to RT and END in healthy young men (Fig. 4). These novel findings suggest that individuals of a certain genotype adapt better to one form of chronic exercise compared to another. Thus, depending on the training objective (*e.g.* strength gains or increased cardiovascular fitness), these findings suggest that exercise may soon be prescribed on an individual basis to optimise health/physical performance benefits.

Our RT and END programmes each lasted four weeks, which is sufficient time to induce gains in muscle strength (Moritani and deVries 1979; Seynnes et al. 2007; Buckthorpe et al. 2015) and cardiovascular fitness (Dunham and Harms 2012; Murias et al. 2016; Dawson et al. 2021). In our cross-over designed study, we observed an increase in MVIC force after RT but not END, while END improved V̇O_2peak_ but not MVIC force, which is in concordance with a 12-week cross-over study (Marsh et al. 2020). Importantly, after our three-week washout period, both MVIC force (following RT) and V̇O_2peak_ (following END) did not differ from pre-training values, thus demonstrating that the three-week detraining period was sufficient for post-training values to return to baseline (Fig. 2). Regarding the first aim of our study, we found that individual adaptations to both modes of exercise training were unrelated (Fig. 3). In other words, a high response to one exercise training modality did not relate to the adaptation to the other exercise modality. This observation reinforces a previous study, which compared both modes of exercise after a longer training duration (*i.e.* 12 weeks) in pairs of male and female same-sex monozygotic and dizygotic twins (Marsh et al., 2020). Despite substantial differences in methodology in the study by Marsh et al. (2020) (*e.g.* RT intensity; mix of upper- and lower-body exercises; 1-RM test to determine maximal strength; END incorporated both running and cycling; and V̇O_2peak_ was performed on a treadmill), both studies demonstrated that intra-individual differences in the adaptation to RT and END were unrelated, whether the training was performed over just four weeks or for three months.

A unique aspect of our study was to explore whether SNPs within specific genes were associated with intra-individual differences in the adaptations to RT *versus* END. Interestingly, having two *VEGFA* rs2010963 G-alleles appeared to facilitate a more favourable adaptation to RT than END, while also providing an advantage over C-allele carriers regarding RT (Fig. 4). While both neural and muscle morphological adaptations contribute to strength gains following RT (Del Vecchio et al. 2019), a significant proportion of strength gains remains unexplained (Erskine et al. 2010b). Importantly, more than 80% force produced by a skeletal muscle fibre is transmitted laterally to the extracellular matrix (ECM) (Ramaswamy et al. 2011), and a RT-induced increase in this force transfer (*e.g.* by effectively splitting the single muscle fibre up into multiple force-generating units) may help to explain the larger gains in muscle strength than size typically seen following RT studies (Erskine et al. 2011). Matrix metalloproteinases (MMPs) play a crucial role in remodelling of the ECM by degrading various collagenous and non-collagenous ECM proteins to maintain tissue homeostasis (Medley et al. 2003), thus potentially influencing lateral force transmission within the muscle. The genes encoding MMPs have previously been associated with several musculoskeletal injuries (Posthumus et al. 2012). Specifically, several SNPs within the matrix metallopeptidase 3 (*MMP3*) gene have been associated with the risk of chronic Achilles tendinopathy (Gibbon et al. 2017) and anterior cruciate ligament rupture (Lulińska-Kuklik et al. 2019). More recently, we showed that academy soccer players with at least one copy of the *MMP3* rs679620 T-allele had more severe injuries, and those with both copies of the *VEGFA* rs2010963 C-allele were 10 times more likely to be injured than GG homozygotes (Hall et al. 2021). This latter finding is likely linked to *VEGFA* rs2010963 C-allele carriers having a higher circulating VEGF-A concentration (Schneider et al. 2008). As VEGF-A upregulates MMP3 expression (Wang and Keiser 1998), which compromises ECM homeostasis (Wang and Keiser 1998; Hall et al. 2021), this may prevent *VEGFA* C-allele carriers from forming as many lateral attachments in response to loading. Thus, it is possible that this hypothetical impaired adaptation to RT may explain the smaller strength gains seen in our C-allele carriers compared to GG homozygotes, as well as the ability of our GG homozygotes to preferentially adapt to RT more than END.

While this is the first study to investigate *VEGFA* genotype with regards to inter- and intra-individual adaptations to RT and END, the *ACTN3* rs1815739 SNP has been investigated in numerous studies regarding an association with the inter-individual adaptation to RT (Clarkson et al. 2005; Delmonico et al. 2007; Gentil et al. 2011; Pereira et al. 2013; Erskine et al. 2014) or END (Silva et al. 2015; Papadimitriou et al. 2019; Romero-Blanco et al. 2020), but this is the first time this SNP has been investigated with regards to the intra-individual adaptations to *both* RT and END in the same participants. Regarding the adaptation to RT alone, previous studies having reported either a beneficial effect of the R-allele (Delmonico et al. 2007; Gentil et al. 2011; Pereira et al. 2013), the X-allele (Clarkson et al. 2005), or no association (Erskine et al. 2014), while the adaptation to END alone is equally unclear (Silva et al. 2015; Papadimitriou et al. 2019; Romero-Blanco et al. 2020). Differences in study design (including population investigated, training intensity, volume and duration) may provide some explanation for the equivocal findings but our within groups cross-over design allowed us to investigate a different question, *i.e.* if individuals with one or two R-alleles adapted better to RT than END, and whether XX homozygotes (α-actinin-3 deficient) would adapt better to END than RT. Although we did not observe any genotype × exercise modality interaction, there was a non-significant tendency (*P* = 0.07) for a genotype effect, suggesting *ACTN3* XX homozygotes tended to adapt better than R-allele carriers to both END and RT. Since elite endurance athletes have a higher frequency distribution of the *ACTN3* XX genotype (Yang et al. 2003), and *ACTN3* XX homozygotes have a greater baseline and improvement in V̇O_2peak_ compared to individuals with *ACTN3* RX and RR genotypes (Kikuchi et al. 2016), a tendency for a change in V̇O_2peak_ following END in XX homozygotes was perhaps unsurprising. However, a tendency for XX homozygotes to adapt better to RT compared to R-allele carriers was unexpected, especially considering the greater composition of type I skeletal muscle fibres in XX homozygotes (Vincent et al. 2007) and the smaller, weaker, and less powerful characteristics of type I compared to type II skeletal muscle fibres (Gilliver et al. 2009). In an earlier study, however, young untrained women of *ACTN3* XX genotype demonstrated a greater improvement in MVIC strength after RT compared to their RR counterparts (Clarkson et al. 2005), while in another study, older women of *ACTN3* RR genotype increased power after RT more than their female XX homozygote counterparts (Delmonico et al. 2007). Nevertheless, it should be emphasised that our tendency for a main effect was non-significant, and this lack of association between ACTN3 genotype and adaptation to RT is in accordance with our previous work, where we demonstrated differences in muscle size, strength and power between XX homozygotes and R-allele carriers in the untrained state but, crucially, no genotype-dependent difference in the trainability to RT (Erskine et al. 2014). Thus, in contrast to young (Clarkson et al. 2005) and older (Delmonico et al. 2007) women (raising the possibility of a sex- and ageing-specific *ACTN3* genotype interaction with RT adaptation), we have shown in two independent studies that the *ACTN3* rs1815739 SNP does not appear to affect the adaptation to RT in young, healthy men.

The third and final SNP we investigated in this study was *NOS3* rs2070744, which has previously been associated with elite power athlete status (Gómez-Gallego et al. 2009) and superior sprint/power performance (Murtagh et al. 2020). These associations may be due to reduced promoter activity of the *NOS3* C-allele (Miyamoto et al. 2000), inhibiting *NOS3* transcription and limiting NO production (Nakayama et al. 2000). As NO can augment myoblast fusion (Long et al. 2006), this SNP has the potential to influence muscle size and therefore strength and power. However, our study is the first to investigate an association of this SNP with the intra-individual adaptations to RT and END in terms of changes in strength and V̇O_2peak_, respectively, in the same homogenous group of individuals. Although not statistically significant (*P* = 0.07), there was a tendency for a genotype × exercise modality interaction, suggesting that C-allele carriers had the capacity to adapt better to RT than they did to END, and also better to RT than their TT homozygote counterparts. These findings are contrary to our hypothesis but, as part of this same project, we recently reported an association of this SNP with the intra-individual adaptations to RT and END in terms of vascular function (Dawson et al. 2021). We found that *NOS3* C-allele carriers tended to show an improvement in vascular function after RT but not END, which we speculated may be linked to transmural pressure, thought to control vascular function during RT (Atkinson et al. 2015). This may activate *NOS3* transcription independently of shear stress, which is thought to regulate vascular function during END. Thus, the mode of exercise (RT over END) may have countered the reduced promoter activity of the C-allele on *NOS3* transcription. Although our study was not able to elucidate how this effect on vascular function may be related to the tendency for a similar C-allele advantage regarding strength gains following RT, it is possible that both are related to transmural pressure that is specific to RT, *i.e.* influencing endothelial function directly and muscle strength indirectly (the latter potentially by increasing myoblast fusion within the vascularised muscle).

### Limitations

To investigate the intra-individual differences in the adaptations to RT *vs*. END, we used a robust cross-over design study. However, several limitations are acknowledged, which could limit the generalisation of our results to other populations. For example, we decided to focus on healthy, young men to keep our group homogeneous (thus, maximising the signal-to-noise ratio), but it is not known whether we would have seen similar results in women, or older/diseased individuals. Furthermore, although our sample size (*n* = 30) was large for a cross-over training intervention study, it may be considered modest for a genetics study. Nonetheless, it was larger than our a priori estimated minimal sample size (*n* = 28) required to detect a genotype × training modality interaction and, after controlling for multiple comparisons, we found significant associations with one of the three SNPs we investigated. Moreover, a post hoc power analysis revealed that our *VEGFA* genotype × training modality interaction achieved 82% power, thus confirming that our study was indeed statistically powered to detect two-way interactions. Nevertheless, independent groups should attempt to replicate our findings in larger cohorts from the same and different populations. Finally, our candidate gene approach isolated three SNPs of interest, while genome-wide association studies (GWAS) are able to investigate hundreds or thousands of SNPs simultaneously. However, unlike GWAS, our approach was hypothesis-driven and based on the evidence that these SNPs have previously been associated with exercise training adaptations and/or elite athlete status. Further, our methodology might be considered more appropriate for smaller, laboratory-controlled studies with precise phenotype measures such as this one, while GWAS technology is considerably more expensive and usually requires hundreds of samples.

## Conclusion

The intra-individual adaptations to short-term RT and END in young, healthy men varied considerably but were unrelated. A variant of the *VEGFA* gene was linked to both the intra- and inter-individual adaptation to RT and END, *i.e. VEGFA* rs2010963 GG homozygotes improved their maximum strength after RT more than their cardiovascular fitness after END, and more than their *VEGFA* C-allele carrier counterparts’ adaptations to RT. This study is the first to provide evidence that genetic variation might help explain the intra-individual adaptation to short-term RT and END in terms of genotype-specific adaptations in strength and cardiovascular fitness, respectively. As muscular strength and cardiovascular fitness are both independent risk factors associated with all-cause mortality, these findings have important implications for personalising exercise prescription to optimise health.

## Data Availability

The data that support the findings of this study are available from the corresponding author upon reasonable request.

## Abbreviations

*ACTN3*: α-Actinin-3 gene
1-RM: Single repetition maximum
ANOVA: Analysis of variance
CPET: Cardiopulmonary exercise testing
ECM: Extracellular matrix
END: Endurance training
eNOS: Endothelial nitric oxide synthase
FDR: False discovery rate
HR: Heart rate
HR_max_: Maximal heart rate
IKD: Isokinetic dynamometer
KE: Knee extension
MMPs: Matric metalloproteinases
MVIC: Maximum voluntary isometric contraction
NO: Nitric oxide
*NOS3*: Nitric oxide synthase 3 gene
RER: Respiratory exchange ratio
RT: Resistance training
SNP: Single-nucleotide polymorphism
SS: Steady-state
*VEGFA*: Vascular endothelial growth factor-A gene
V̇O_2_: Oxygen uptake
V̇O_2peak_: Peak oxygen uptake
